# Global research trends in biliary atresia-related liver fibrosis: a bibliometric analysis (2000–2024)

**DOI:** 10.3389/fped.2026.1546277

**Published:** 2026-03-16

**Authors:** Youcheng Zhang, Bing Li, Shunlin Xia, Ting Wang

**Affiliations:** Department of Pediatric Surgery, Huai’an Maternal and Child Health Care Hospital Affiliated to Yangzhou University, Huai’an, China

**Keywords:** bibliometrics, biliary atresia, CiteSpace, liver fibrosis, pediatrics

## Abstract

**Background:**

Biliary atresia (BA) is a severe neonatal liver disorder that can progress to liver fibrosis and eventual failure. This bibliometric study evaluates global research on BA-related liver fibrosis from 2000 to 2024, highlighting emerging trends and key contributions.

**Methods:**

Data were retrieved from the Web of Science Core Collection, focusing on original articles and reviews. Bibliometric tools were employed to assess publication trends, citation impact, and research collaboration.

**Results:**

A total of 589 publications were identified from 2000 to 2024. Publication output showed relative stability from 2000 to 2014 (223 publications), followed by a marked increase to 296 publications during 2015–2022, with continued growth in 2023 (39 publications) and 2024 (31 publications) [*χ*^2^(1) = 113.28, *p* < 0.001 for the 2000–2014 vs. 2015–2022 comparison]. The leading contributors were China, the USA, and Japan. Notable institutions included Chulalongkorn University, Fudan University, and University of Cincinnati. Author analysis identified a small group of prolific researchers with high publication counts and H-indices, such as Yong Poovorawan (40 publications, H-index 12) and Michael Davenport (24 publications, H-index 15), indicating substantial research impact. Core journals in the field included Pediatric Surgery International, Journal of Pediatric Surgery, and Journal of Pediatric Gastroenterology and Nutrition, all of which demonstrated high publication volumes and impact factors. Keyword co-occurrence analysis revealed research clusters around pathogenesis, management, molecular mechanisms, non-invasive biomarkers, and imaging techniques. Co-citation analysis highlighted early diagnosis, surgical outcomes, and pathogenesis as central research themes. Future trends suggest a growing focus on non-invasive diagnostics, molecular mechanisms, and international collaboration, with keywords such as “pathogenesis,” “outcome,” “elasticity imaging techniques,” and “shear wave elastography” showing citation bursts.

**Conclusion:**

Research on BA-related liver fibrosis has significantly increased, with key contributions from leading countries, institutions, and authors. Core journals have been instrumental in shaping the research discourse. This study provides valuable insights into current research trends and future directions, emphasizing the importance of interdisciplinary collaboration in advancing the understanding and treatment of BA-related liver fibrosis.

## 1 Introduction

Biliary atresia (BA) is a severe neonatal liver disorder characterized by the progressive destruction of extrahepatic bile ducts, resulting in bile stasis, liver fibrosis, and, if not managed promptly, cirrhosis and liver failure (1). BA is the leading indication for liver transplantation in children. Liver fibrosis in BA is a key determinant of patient prognosis, with significant implications for long-term outcomes (2, 3). Despite advancements in early diagnosis and therapeutic interventions, such as the Kasai portoenterostomy, liver transplantation remains the definitive treatment for end-stage liver disease in patients with BA. Therefore, understanding the pathophysiology of liver fibrosis in BA is critical for improving diagnosis, treatment strategies, and patient outcomes (4).

Recent research on liver fibrosis related to BA has expanded, particularly with the advancement of molecular techniques, imaging technologies, and non-invasive diagnostic methods (5, 6). Although considerable progress has been made in elucidating the mechanisms of liver fibrosis, the global research landscape remains fragmented (7–9). While various countries and institutions have contributed to this field, a comprehensive overview of trends, key players, and emerging research hotspots is still lacking. This study aimed to fill this gap by conducting a bibliometric analysis of global research on liver fibrosis in BA from 2000 to 2024. Bibliometric analysis is a powerful tool for mapping scientific literature, identifying major contributors, research trends, and the evolution of the field over time (10–12). By analyzing publication patterns, citation bursts, and co-citation networks, we aimed to provide a clearer understanding of the development of research on BA-related liver fibrosis, highlight areas of significant focus (particularly the ongoing efforts to monitor liver fibrosis in BA), and identify directions for future research. Furthermore, this study emphasizes the role of core journals and international collaborations in advancing the field and identifies emerging areas, such as non-invasive biomarkers, imaging techniques, and molecular mechanisms, that are expected to influence future research (13, 14).

## 2 Method

### 2.1 Data source

The data for this study were sourced from the Web of Science Core Collection (WOSCC). As a globally recognized database hosting numerous influential and high-quality journals, Web of Science is extensively utilized in bibliometric research (15, 16).

### 2.2 Data retrieval and extraction strategies

**Figure 1:**
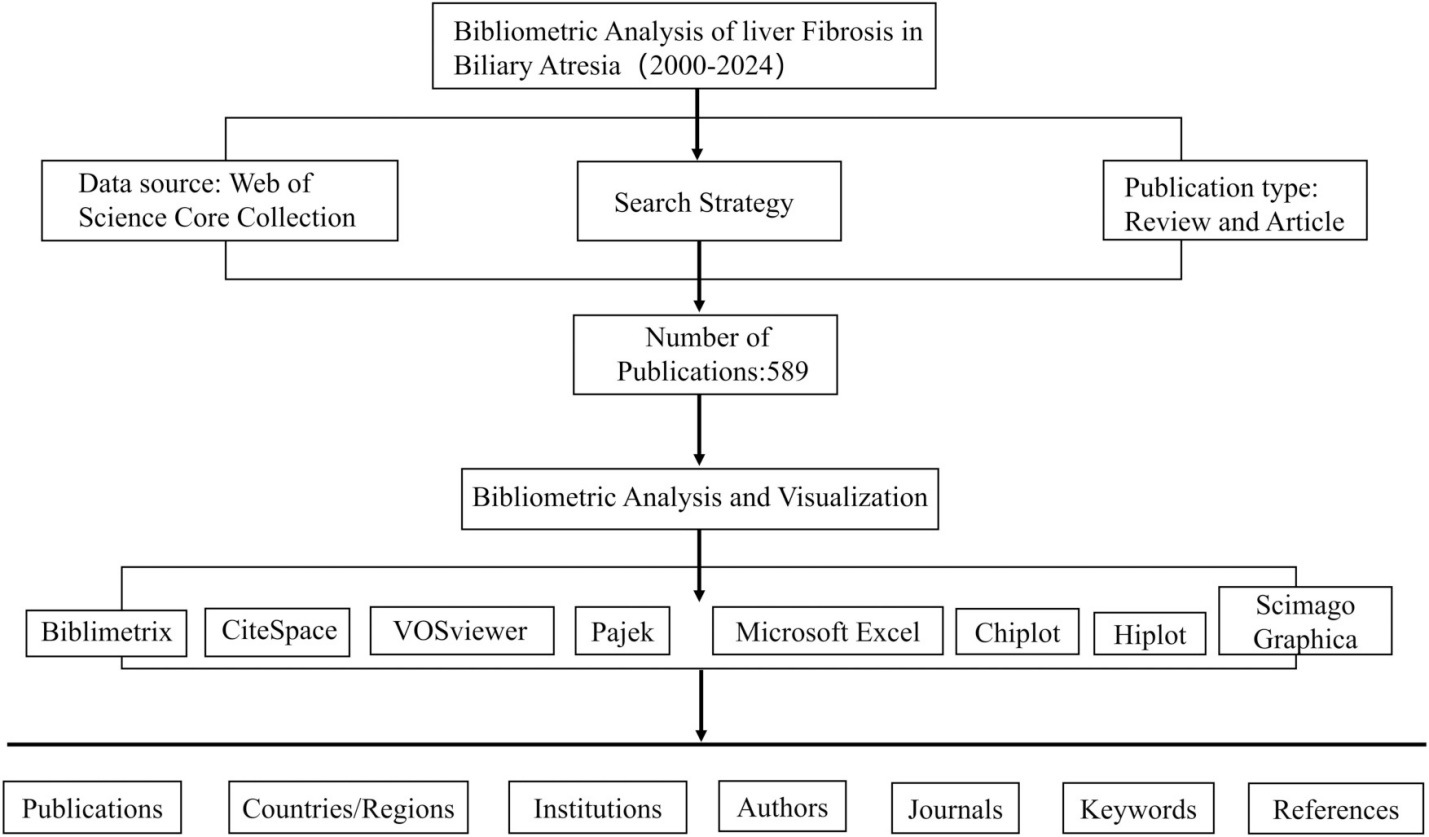
Flowchart illustrating the data retrieval, selection, and analysis process for the bibliometric study on biliary atresia-related liver fibrosis (2000–2024).

The search strategy is outlined in Table 1. Only original articles and reviews were included, while proceeding papers, meeting abstracts, early access items, editorials, retracted publications, letters, and book chapters were excluded. Articles published between 2000 and 2024 that met these criteria were selected. Key details from each selected article—including title, authors and their affiliations, abstract, keywords, references, journal name, country/region, and publication year—were downloaded and exported as plain text (TXT) (Figure 1).

**Table 1:** Search strategy used in the Web of science core collection for retrieving publications on biliary atresia-related liver fibrosis (2000–2024).

Step	Query	Result
#1	(((((((([TI = (biliary atresia)] OR TI = (Atresia, Biliary)) OR TI = (Idiopathic Extrahepatic Biliary Atresia)) OR TI = (Familial Extrahepatic Biliary Atresia)) OR TI = (Biliary Atresia, Extrahepatic)) OR TI = (Atresia, Extrahepatic Biliary)) OR TI = (Extrahepatic Biliary Atresia)) OR TI = (Intrahepatic Biliary Atresia)) OR TI = (Atresia, Intrahepatic Biliary)) OR TI = (Biliary Atresia, Intrahepatic)	3,817
#2	((([TS = (Cirrhosis, Liver)] OR TS = (Hepatic Cirrhosis)) OR TS = (Cirrhosis, Hepatic)) OR TS = (Fibrosis, Liver)) OR TS = (Liver Fibrosis)	1,51,887
#3	#1 AND #2	745
#4	#3 AND Publication Date: 2000-01-01 to 2024-09-29	656
#5	#4 AND Proceeding Paper or Meeting Abstract or Early Access or Editorial Material or Retracted Publication or Letter or Book Chapters (Exclude-Document Types)	593
#6	#5 AND English (Languages)	589

### 2.3 Bibliometric and visual analysis

We utilized the R-based bibliometric analysis software Bibliometrix to process and analyze the literature data, importing TXT files for further organization (17). Additionally, we employed Bibliometrix, CiteSpace (version 6.2.R4), VOSviewer (version 1.6.19), Pajek (version 5.19), Scimago Graphica, Hiplot, Chiplot, and Microsoft Office Excel 2019 for visualization and quantitative analysis of the collected literature, including coupling and co-occurrence analyses.

## 3 Results

### 3.1 Trends and influential publications in biliary atresia and liver fibrosis research

**Figure 2:**
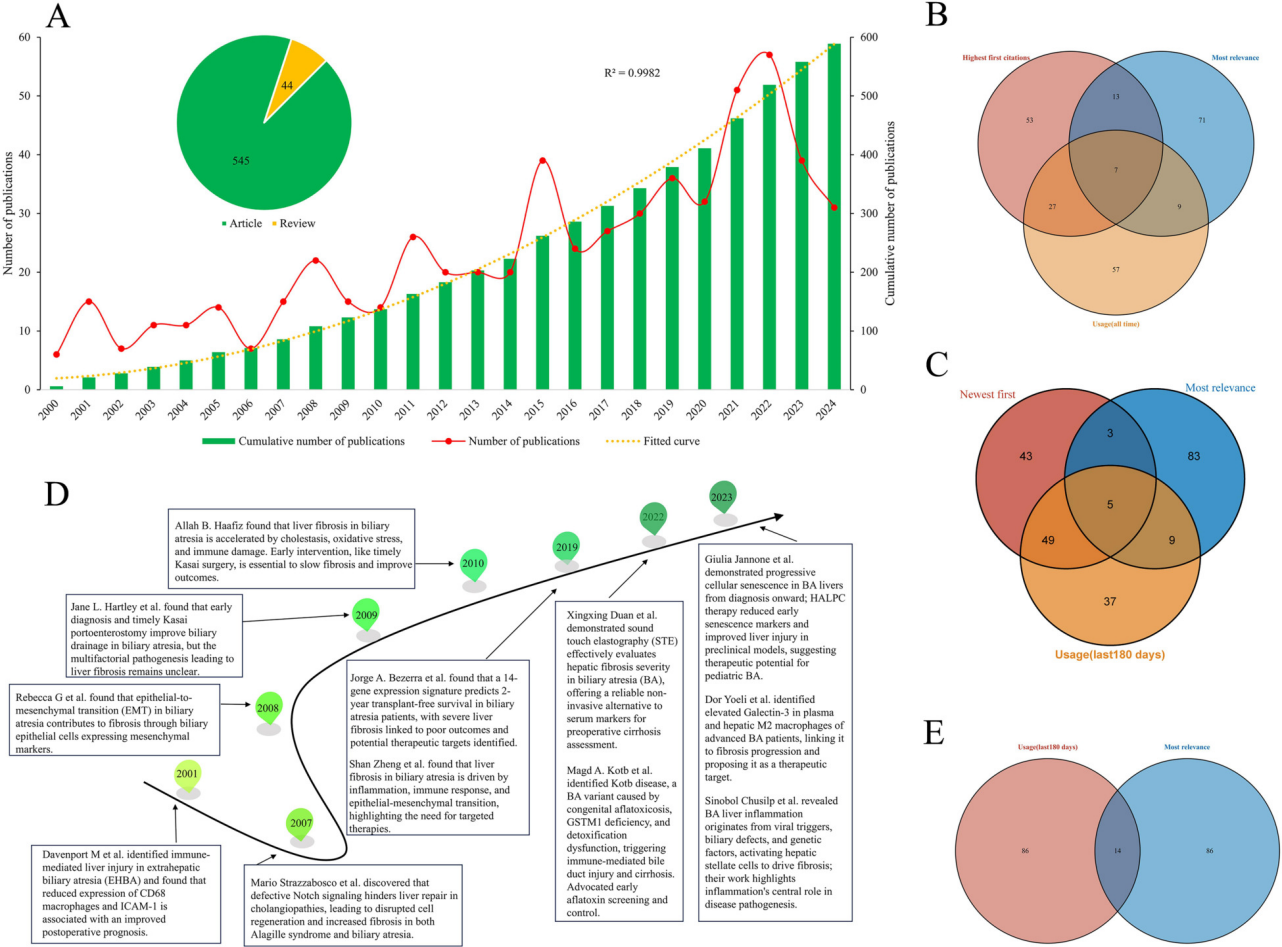
Trends in publications on biliary atresia and liver fibrosis from 2000 to 2024, including annual and cumulative counts, overlaps in top-cited papers, and key research milestones. **(A)** Annual and cumulative publication trends from 2000 to 2024. Green bars show cumulative publications, and the red line represents yearly publications. The pie chart categorizes 545 articles and 44 reviews. The fitted curve (yellow dotted line) has an *R*^2^ of 0.9982. **(B)** Venn diagram displaying the overlap of the top 100 papers ranked by high first citation, most relevance, and all-time usage. Seven papers are common across all categories. **(C)** Venn diagram displaying the overlap of the top 100 papers ranked by newest first citation, most relevance, and usage in the last 180 days. Five papers are common across all categories. **(D)** Timeline summarizing key findings from the 12 overlapping papers identified in Panel B and C, showing research progress from 2001 to 2019. **(E)** Venn diagram highlighting the overlap of the top 100 papers ranked by most relevance and usage in the last 180 days, with 14 papers in common.

An overview of research publications on biliary atresia and liver fibrosis from 2000 to 2024 is shown (Figure 2). The annual and cumulative publication trends are illustrated (Figure 2A). From 2000 to 2014, publication counts remained stable at 223. A marked surge to 296 publications occurred between 2015 and 2022, peaking from 2017 to 2020, with continued growth into 2023 (39 publications) and 2024 (31 publications), bringing the total to 589 publications across the full period. The robust upward trend is evidenced by a fitted curve (*R*^2^ = 0.9982); to further quantify this accelerating growth pattern, we supplemented a quadratic function fitting analysis, with the regression equation being *y* = 0.8562*x*^2^ + 1.4471*x* + 17.046 (where *x* represents the time offset from 2000, and y denotes the annual number of publications). This quadratic model better captures the “pre-2015 stability and post-2015 acceleration” trend consistent with the two-phase comparison, further supporting the reliability of the observed growth dynamics. Overall, 589 publications (545 articles, 44 reviews) were analyzed. A chi-square test comparing Poisson rates between periods (2000–2014 vs. 2015–2022) yielded *χ*^2^(1) = 113.28 (*p* < 0.001), confirming a statistically significant acceleration in publication output post-2015. A Venn diagram showing the overlap of the top 100 papers ranked by highest first citation, most relevance, and all-time usage is presented (Figure 2B). Seven papers were common across all three categories, indicating consistent recognition and relevance.

A Venn diagram showing the overlap of the top 100 papers ranked by newest first citation, most relevance, and usage in the last 180 days is presented (Figure 2C). Five papers common to all three categories not only demonstrate consistent recognition and relevance but also highlight cutting-edge advancements integrated with current thematic priorities and emerging dynamics of scholarly engagement.

A timeline summarizing key findings from the seven overlapping papers is provided (Figure 2D). The timeline highlights significant milestones in understanding liver fibrosis mechanisms and therapeutic approaches for biliary atresia, covering immune-mediated injury, epithelial-to-mesenchymal transition, defective signaling pathways, and gene expression signatures from 2001 to 2019.

The overlap between the top 100 papers ranked by most relevance and usage in the last 180 days is highlighted (Figure 2E). Fourteen papers were common to both categories, suggesting these publications are both highly impactful and currently of considerable interest (Table 2).

**Table 2:** Fourteen papers appear in both top relevance and usage categories.

Author	Title	Year	Main focus of the literature
Yoeli et al. (40)	Galectin-3 in biliary atresia and other pediatric cholestatic liver diseases.	2023	Explores the role of Galectin-3 in biliary atresia (BA) and other pediatric cholestatic liver diseases, with a particular focus on its association with liver fibrosis progression.
Jannone et al. (39)	Senescence and senotherapies in biliary atresia and biliary cirrhosis.	2023	Explores cellular senescence in biliary atresia and biliary cirrhosis, while evaluating the effectiveness of senotherapies in a preclinical model.
Pierroet al. (5)	Development of liver infammatory injury in biliary atresia: from basic to clinical research.	2023	Examines liver inflammatory injury and the rapid fibrosis mechanisms in biliary atresia, with a focus on immune response and cytokine involvement.
Kotb et al. (38)	Congenital aflatoxicosis, mal-detoxification genomics & ontogeny trigger immune-mediated Kotb disease biliary atresia variant SANRA compliant review.	2022	Explores Kotb disease, a biliary atresia variant driven by congenital aflatoxicosis and GSTM1 deficiency, resulting in immune-mediated liver damage, fibrosis, and accelerated cirrhosis.
Duan et al. (37)	Sound touch elastography for assessing cirrhosis preoperatively in infants with biliary atresia: Comparison with serum fibrosis biomarkers.	2022	Evaluates sound touch elastography (STE) for preoperative cirrhosis assessment in biliary atresia infants, highlighting its strong correlation with fibrosis stages and superior diagnostic accuracy compared to serum biomarkers.
Du et al. (58)	Hemodynamic analysis of hepatic arteries for the early evaluation of hepatic fibrosis in biliary atresia.	2021	Utilizes computational fluid dynamics (CFD) to analyze hepatic artery hemodynamics in biliary atresia, revealing significant fibrosis progression and emphasizing CFD's potential as a non-invasive diagnostic tool.
Ueno et al. (56)	Impact of serum autotaxin level correlating with histological findings in biliary atresia.	2021	Explores the relationship between serum autotaxin levels and liver fibrosis in biliary atresia, emphasizing autotaxin's potential as a non-invasive marker for fibrosis assessment.
Chung et al. (59)	Biomarkers for the diagnosis and post-Kasai portoenterostomy prognosis of biliary atresia: a systematic review and meta-analysis.	2021	Reviews biomarkers such as MMP-7, IL-33, and GGT for diagnosing biliary atresia, and emphasizes APRi (Aspartate Aminotransferase to Platelet Ratio Index)'s role in predicting liver fibrosis and cirrhosis after Kasai portoenterostomy.
Luo et al. (36)	Gene Expression Signatures Associated With Survival Times of children With Biliary Atresia Identify Potential Therapeutic Agents.	2021	Identifies a 14-gene expression signature that predicts 2-year transplant-free survival in patients with BA and investigates N-acetyl-cysteine (NAC) as a potential treatment to reduce fibrosis and enhance survival.
Ueno et al. (60)	Serum Mac-2-binding protein (M2BPGi) as a marker of chronological liver fbrosis in biliary atresia patients with cirrhosis.	2019	Evaluates serum Mac-2-binding protein glycosylation isomer (M2BPGi) as a marker for monitoring liver fibrosis progression in pediatric patients with BA, emphasizing its role in predicting decompensated cirrhosis and optimizing liver transplant timing.
Zheng et al. (26)	Liver fbrosis in biliary atresia.	2018	Reviews the mechanisms of liver fibrosis in biliary atresia, focusing on ductal plate malformation, immune responses, and epithelial–mesenchymal transition (EMT), which plays a key role in fibrosis development.
Clemente et al. (61)	Intra-Hepatic bile duct primary cilia in biliary atresia.	2021	Explores the abnormalities of intra-hepatic bile duct primary cilia in biliary atresia and their association with clinical outcomes.
Karakoyun et al. (62)	Infants with extrahepatic biliary atresia: Effect of follow-up on the survival rate at Ege University Medical School transplantation center.	2017	Reviews the outcomes of infants with extrahepatic biliary atresia at Ege University, comparing survival rates after Kasai portoenterostomy and liver transplantation, emphasizing the importance of early diagnosis and follow-up for improved survival.
Hartley et al. (21)	Biliary atresia.	2009	Provides an in-depth review of the epidemiology, pathophysiology, diagnosis, and management of biliary atresia, with a focus on liver fibrosis and surgical outcomes.

### 3.2 Countries/regions analysis

**Figure 3:**
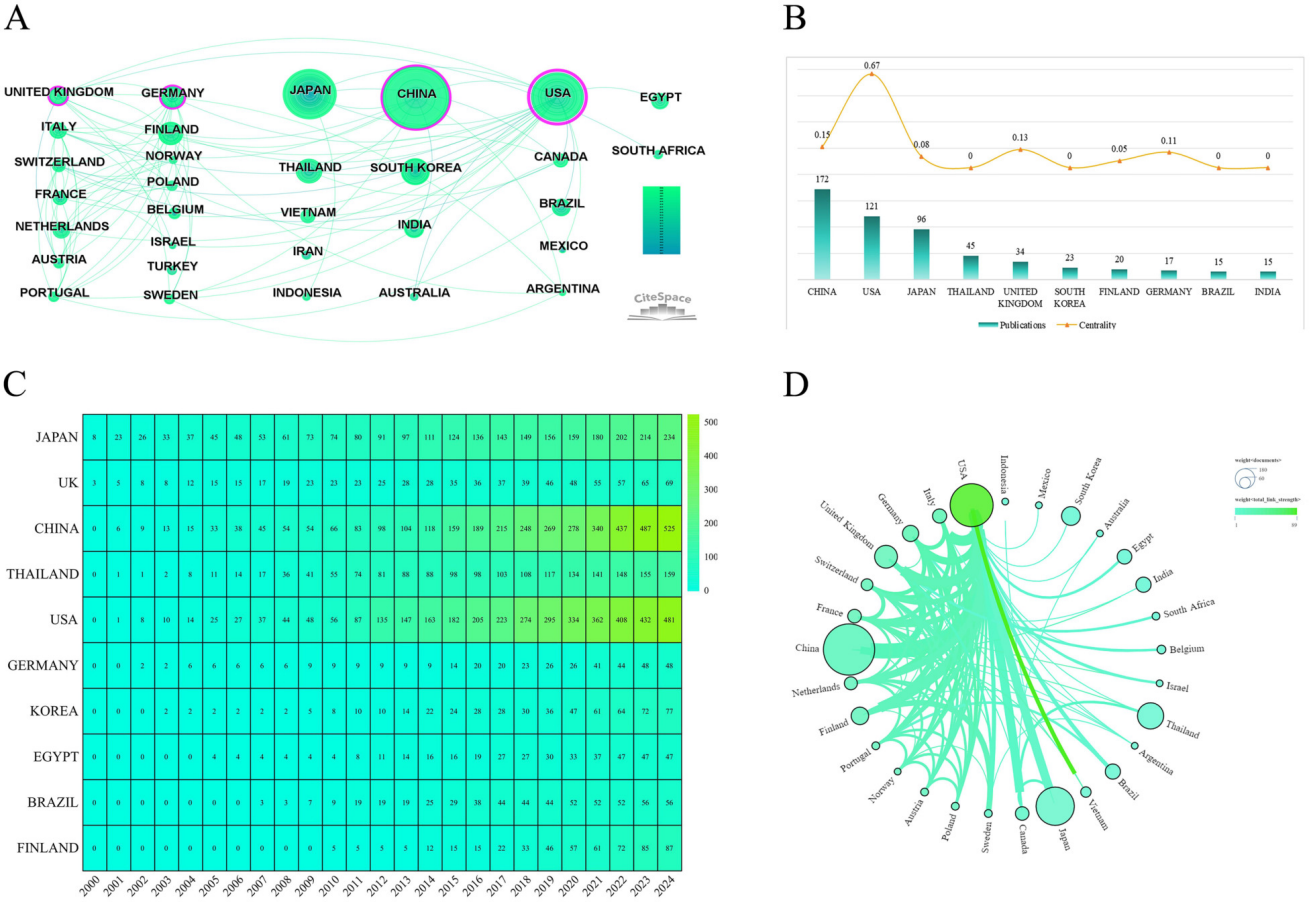
International collaboration and publication trends in liver fibrosis research related to biliary atresia. **(A)** Country collaboration network generated using CiteSpace, with node size representing the number of publications and countries with centrality greater than 0.1 highlighted. **(B)** Publication counts and centrality scores of the top 10 countries in liver fibrosis research related to biliary atresia. **(C)** Heatmap depicting annual publication counts for the top 10 countries based on citation frequency. **(D)** International collaboration network in liver fibrosis research within biliary atresia, where node size indicates the number of publications and line thickness reflects the strength of collaboration.

Figure 3 provides an overview of international collaboration and publication trends in liver fibrosis research related to biliary atresia. Figure 3A shows the country collaboration network generated using CiteSpace. China (172 publications), the USA (121 publications), and Japan (96 publications) are the top contributors. Node size reflects publication volume, with larger nodes indicating greater output. Figure 3B presents the publication counts and centrality scores of the top 10 countries. Countries such as the USA, China, the United Kingdom, and Germany have centrality values greater than 0.1 (USA: 0.67, China: 0.13, United Kingdom: 0.13, Germany: 0.11), indicating their key roles in connecting collaborative networks. Figure 3C shows a heatmap of annual publication counts for the top 10 countries based on citation frequency. The data reveal increasing publication trends for China, the USA, and Japan, demonstrating their continued focus on liver fibrosis research in biliary atresia. Figure 3D illustrates the international collaboration network. Node size represents publication volume, while line thickness indicates collaboration strength. The USA and China are key players with strong international ties, and several European countries, such as Germany and the United Kingdom, also show active participation in collaborative research.

### 3.3 Institutional analysis

**Figure 4:**
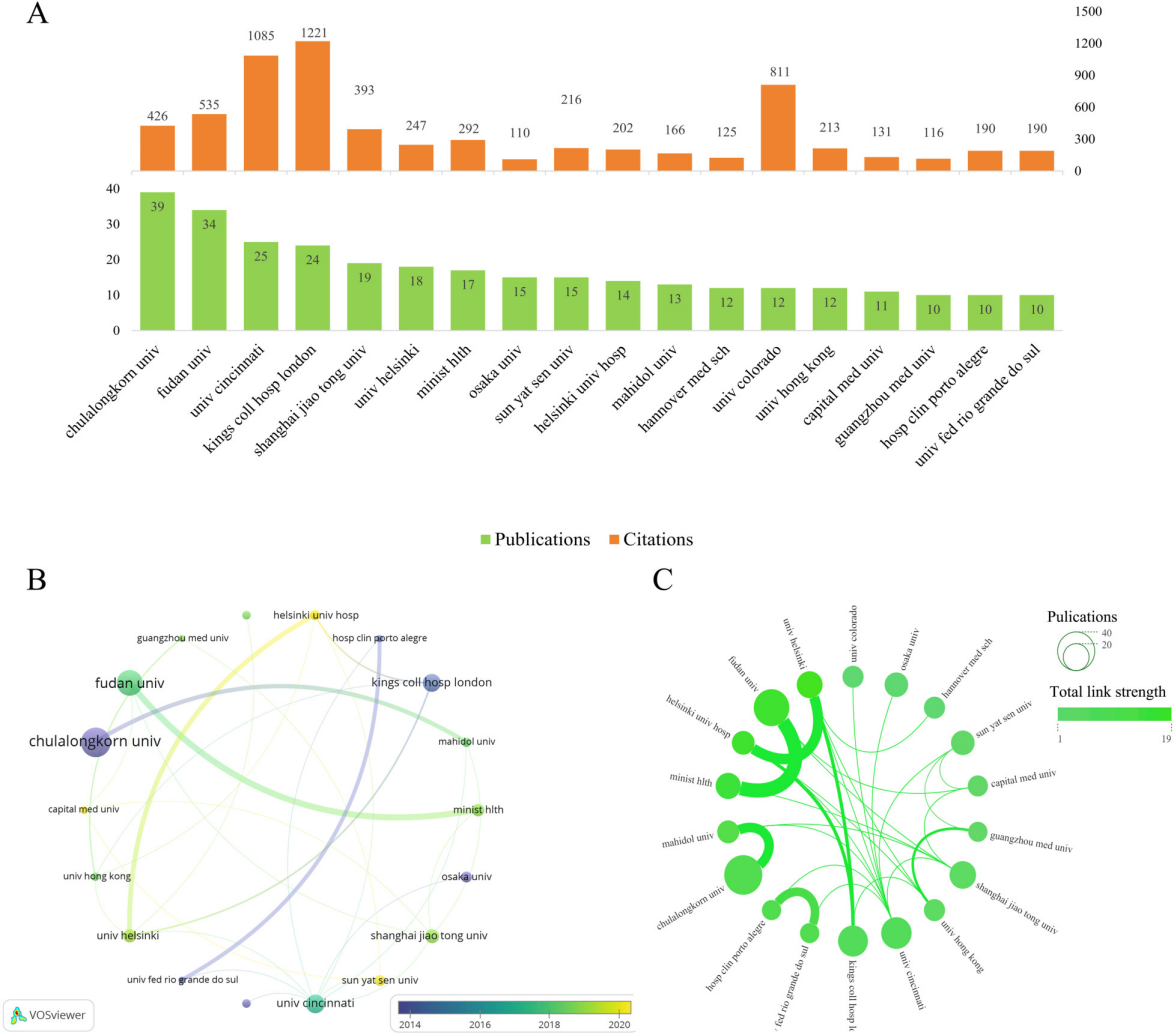
Institutional publication analysis on biliary atresia related liver fibrosis research. **(A)** Bar chart of publication volume and citation counts by institution, Green bars indicate the number of publications, and orange bars indicate citation counts. **(B)** Overlay visualization of institutional publication volume and trends over time (VOSviewer analysis), with 18 institutions with at least 10 publications shown. **(C)** Network visualization of institutional collaboration in biliary atresia fibrosis research.

The institutional analysis comprised 18 nodes and multiple collaborative connections (Figure 4). Over the past decade, 18 institutions have contributed significantly to biliary atresia fibrosis research, with Chulalongkorn University (*n* = 39) leading in the number of publications, followed by Fudan University (*n* = 34) and University of Cincinnati (*n* = 25). In terms of citations, King's College Hospital London ranked highest (*n* = 1221), followed by University of Cincinnati (*n* = 1,085) and University of Colorado (*n* = 811) (Figure 4A). Recent research activity, as shown in Figure 4B, has been concentrated at Sun Yat-sen University, Capital Medical University, Helsinki University Hospital, University of Helsinki, and Shanghai Jiao Tong University, reflecting increasing interest in this field. Collaboration among institutions is primarily organized into three major networks: (1) University of Cincinnati, Children's Hospital of Philadelphia, University of Michigan, Washington University, and Texas Children's Hospital; (2) Fudan University and the Ministry of Health; and (3) Chulalongkorn University and Mahidol University, highlighting key partnerships driving research in this area (Figure 4C).

### 3.4 Authors and co-authors analysis

**Figure 5:**
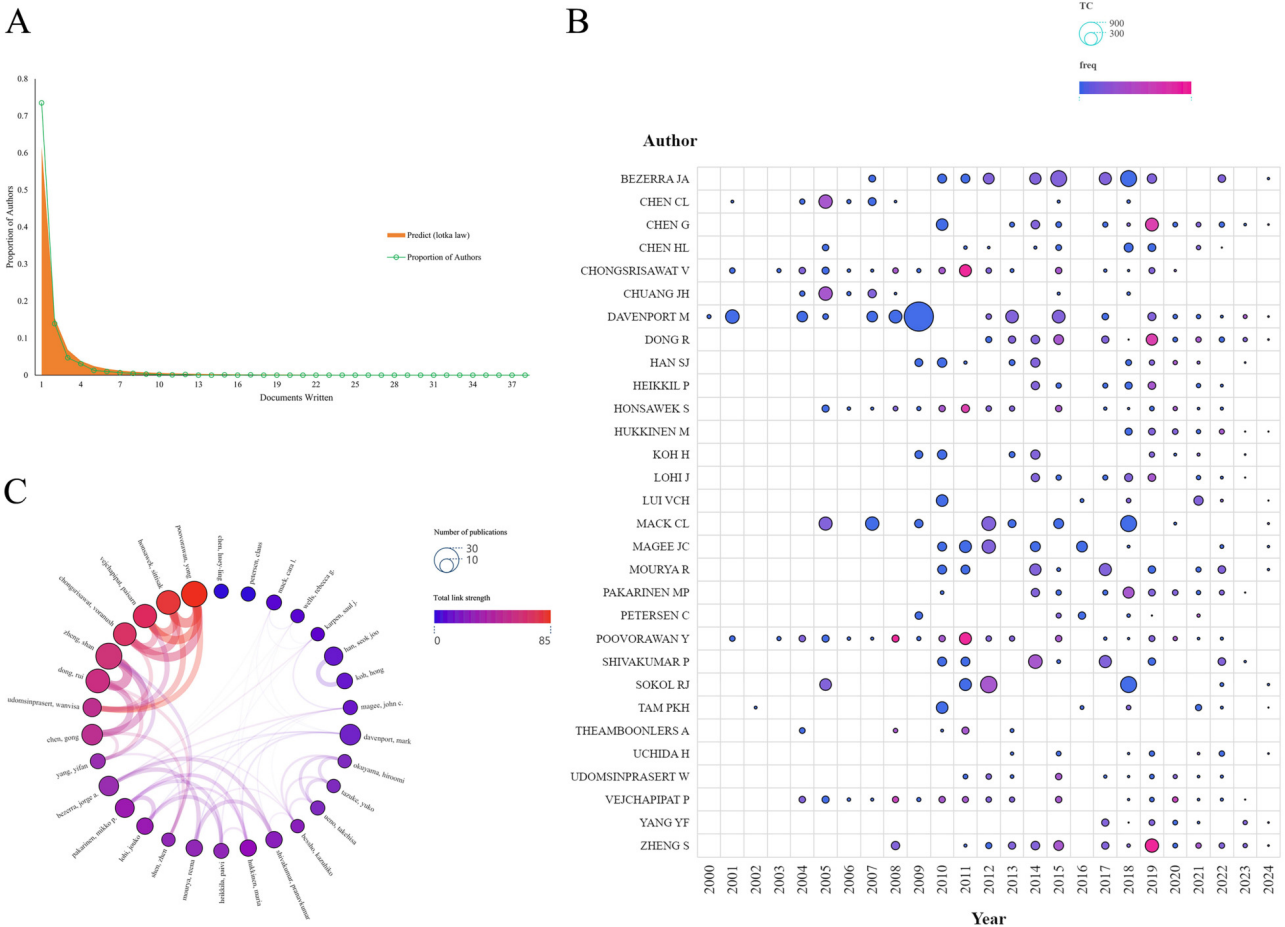
An analysis of author productivity, citation impact, and collaboration in hepatic fibrosis research on biliary atresia. **(A)** Scientific productivity of authors based on Lotka's law. **(B)** A bubble chart created with SCImago displaying the top 30 authors by publication frequency. **(C)** Network visualization of collaborative relationships among authors in biliary atresia-related fibrosis research.

Authorship analysis in hepatic fibrosis research related to biliary atresia reveals concentrated productivity, impact, and collaboration patterns. Lotka's law (Figure 5A) shows that most of the 2,591 authors contributed only once, with 73.5% publishing a single paper, while a small number of prolific authors, such as Poovorawan (40 publications, H-index 12), Davenport (24 publications, H-index 15), and Vejchapipat (33 publications, H-index 10), dominate the field. Davenport leads in citation impact (1,526 citations), followed by Bezerra (960) and Mack (793) (Figure 5B and Table 3). Network analysis highlights two main collaborative groups: Poovorawan Yong, Honsawek Sittisak, Vejchapipat Paisarn, Chongsrisawat Voranush, and Udomsinprasert Wanvisa form a tightly-knit team, while Zheng Shan, Dong Rui, and Chen Gong also collaborate closely (Figure 5C). These clusters emphasize the cohesive efforts and shared expertise driving progress in biliary atresia-related hepatic fibrosis research.

**Table 3:** The top 10 most prolific and highly cited authors in the study of liver fibrosis in biliary atresia.

Author	Count	H-index	Cited Author	Count	H-index
Poovorawan, Y.	40	12	Davenport, M.	1,526	15
Vejchapipat, P.	33	10	Bezerra, J.A.	960	14
Chongsrisawat, V.	32	12	Mack, C.L.	793	9
Zheng, S.	31	15	Kelly, D.A.	737	2
Honsawek, S.	30	10	Hartley, J.L.	651	1
Dong, R.	25	13	Sokol, R.J.	628	8
Davenport, M.	24	15	Zheng, S.	519	15
Chen, G.	20	11	Magee, J.C.	490	7
Bezerra, J.A.	17	14	Shivakumar, P.	476	10
Pakarinen, M.P.	17	11	Karpen, S.J	476	7

### 3.5 Most productive and influential journal analysis

**Figure 6:**
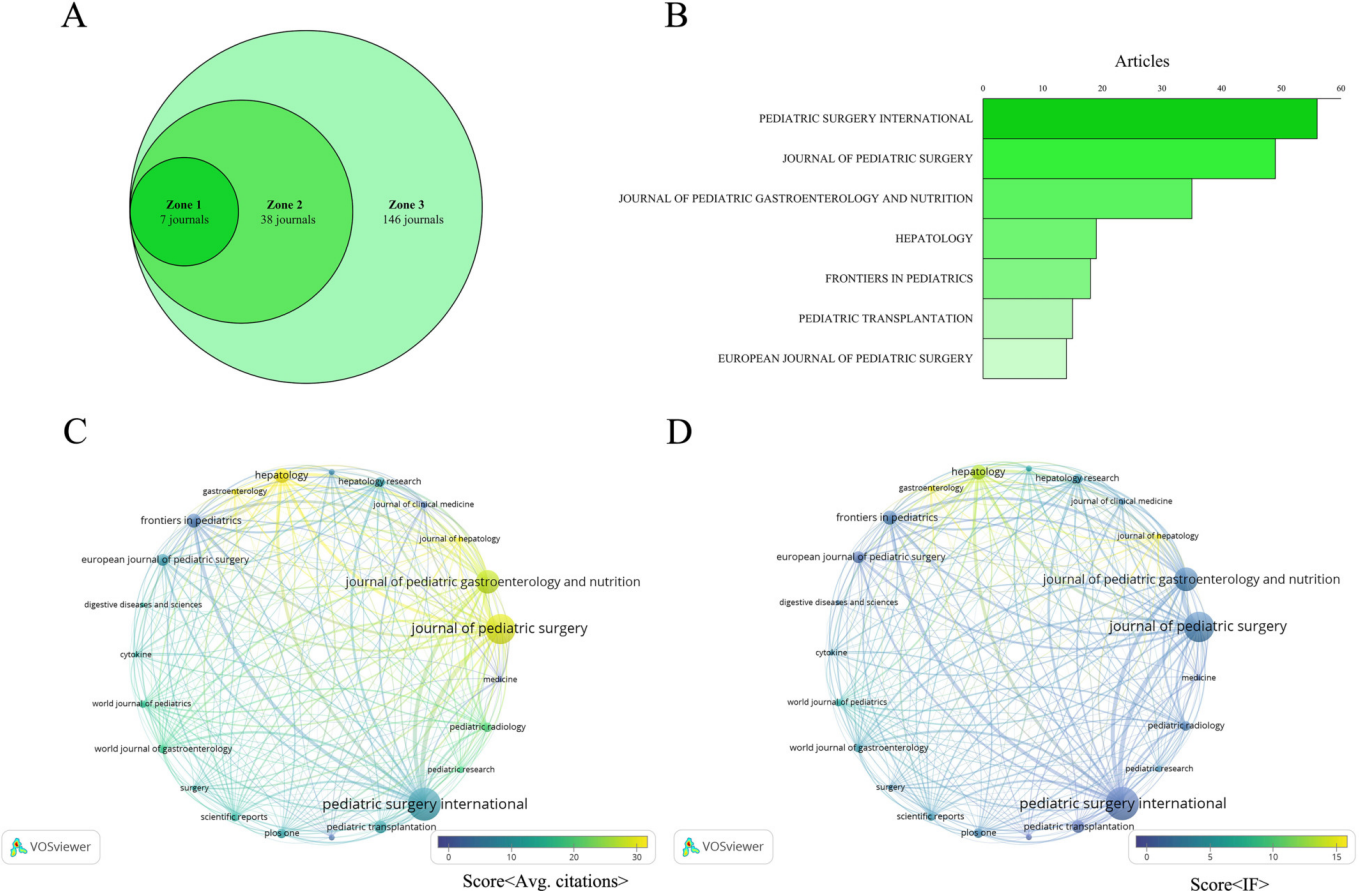
Analysis of journal publication distribution and impact in biliary atresia-related liver fibrosis research. **(A)** Journals distributed by research output under Bradford's Law. **(B)** Publications across seven core journals in Zone 1 by volume. **(C)** Twenty-three journals with at least 5 publications visualized in a VOSviewer co-citation overlay. **(D)** Twenty-three journals visualized in a VOSviewer overlay by impact factor and volume.

In biliary atresia-related hepatic fibrosis research, 191 journals contribute to the field, with research output concentrated in a few core publications, consistent with Bradford's Law. This distribution divides journals into three zones: Zone 1, the core zone, includes 7 key journals; Zone 2 comprises 38 journals; and Zone 3 includes 146 journals. Journals in Zone 1 publish the majority of relevant articles, making them the primary sources of information in this area (Figure 6A).

Among these core journals, Pediatric Surgery International, Journal of Pediatric Surgery, and Journal of Pediatric Gastroenterology and Nutrition stand out not only in publication volume but also in their influence, as indicated by high citation counts and impact factors (Figure 6B). Specifically, Pediatric Surgery International leads with 56 publications, followed by Journal of Pediatric Surgery (49 publications) and Journal of Pediatric Gastroenterology and Nutrition (35 publications), emphasizing their central role in this research domain.

A combined analysis of co-citations and impact factors further underscores the significance of these journals. In the co-citation network of 23 journals with at least five publications, Journal of Pediatric Gastroenterology and Nutrition, Journal of Pediatric Surgery, and Pediatric Surgery International emerge as highly cited and frequently referenced, indicating their wide usage and impact within the field (Figure 6C). Similarly, impact factor analysis of these journals reveals that Pediatric Surgery International, Journal of Pediatric Surgery, and Journal of Pediatric Gastroenterology and Nutrition not only have high publication volumes but also strong impact factors, further highlighting their influence (Figure 6D).

Additional metrics from the top 10 most productive journals reveal that Journal of Pediatric Surgery has the highest total citations (1,377) and an average of 28.10 citations per article, while Hepatology stands out with the highest average citations per article (56.63) and the highest impact factor (IF: 12.9). These metrics underscore the quality and impact of research published in these leading journals, illustrating the significant influence a few key publications have on advancing knowledge in hepatic fibrosis related to biliary atresia (Table 4).

**Table 4:** Top 10 most productive journals in biliary atresia related liver fibrosis field.

Journal	NP	Proportion (%)	TC	Avg. citations	h-index	IF (2023)
PEDIATRIC SURGERY INTERNATIONAL	56	9.51	480	8.57	13	1.5
JOURNAL OF PEDIATRIC SURGERY	49	8.32	1,377	28.10	21	2.4
JOURNAL OF PEDIATRIC GASTROENTEROLOGY AND NUTRITION	35	5.94	931	26.60	18	2.4
HEPATOLOGY	19	3.23	1,076	56.63	14	12.9
FRONTIERS IN PEDIATRICS	18	3.06	74	4.11	5	2.1
PEDIATRIC TRANSPLANTATION	15	2.55	156	10.40	8	1.2
EUROPEAN JOURNAL OF PEDIATRIC SURGERY	14	2.38	118	8.43	7	1.5
PEDIATRIC RADIOLOGY	11	1.87	215	19.55	8	2.1
PLOS ONE	11	1.87	114	10.36	7	2.9
HEPATOLOGY RESEARCH	11	1.87	109	9.91	6	3.9
PEDIATRIC SURGERY INTERNATIONAL	56	9.51	480	8.57	13	1.5

NP, number of Publications; TC, total citations; IF, impact factor.

### 3.6 Keywords analysis of research hotspots

**Figure 7:**
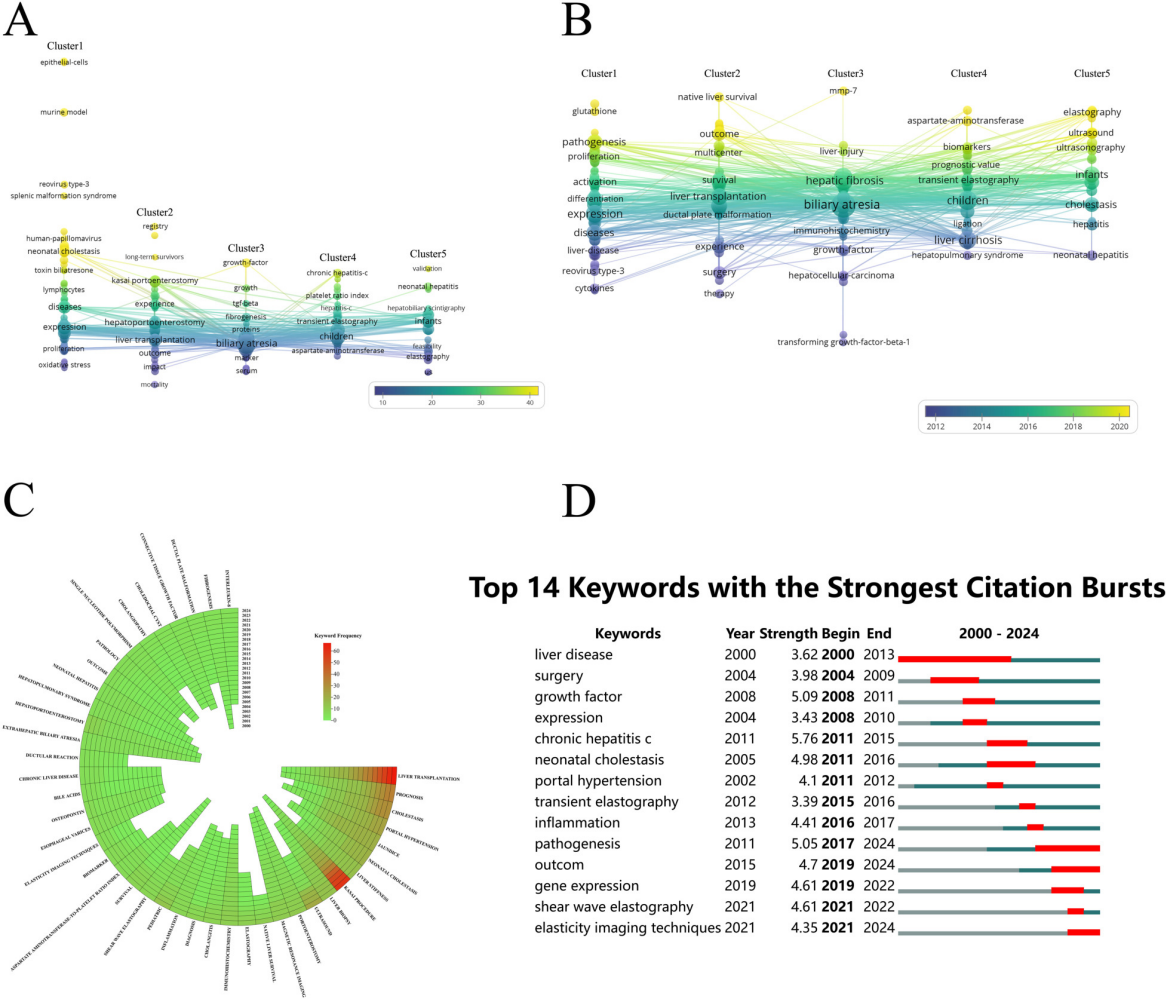
Co-occurrence analysis of global research on hepatic fibrosis in biliary atresia. **(A)** Keyword cluster and overlay map of research hotspots generated with VOSviewer. Colors represent citation frequency, with lighter shades indicating more frequently cited keywords. **(B)** Time-based overlay map of keyword co-occurrence, showing recency of keyword usage with lighter shades indicating more recent appearances, thereby highlighting emerging trends. **(C)** Circular heatmap of the top 50 keywords by frequency, excluding primary terms like “biliary atresia” and “fibrosis” and their synonyms. A total of 42 keywords were selected, with darker shades representing higher frequency in each year, capturing shifts in research focus. **(D)** The top 14 keywords with the strongest citation bursts from 2000 to 2024. The blue line represents the full timeline, while red segments indicate periods of intense citation bursts for each keyword, marking topics that gained rapid research interest over time.

In the co-occurrence analysis, a total of 1,991 keywords were extracted from 589 publications and analyzed using VOSviewer. After removing synonyms and irrelevant terms (e.g., “age,” “years”), 191 keywords with at least 5 co-occurrences were included, revealing several research clusters. As shown in Figure 7A, the keywords were classified into five main clusters, each reflecting a distinct research focus: Cluster 1 centers on pathogenesis, genetic factors, and immune responses in biliary atresia and fibrosis progression, with high-frequency keywords like “epithelial cells” (Avg. citations = 102.11), “murine model” (86.71), and “reovirus type-3” (64.30); Cluster 2 focuses on the management of biliary atresia and fibrosis, including predictors of long-term liver survival and treatment outcomes, featuring terms such as “registry” (51.13), “long-term survivors” (41.67), “single-center” (34.90), and “Kasai portoenterostomy” (34.35); Cluster 3 addresses biliary atresia and hepatic fibrosis through key markers, immune responses, and molecular mechanisms, with keywords like “growth factor” (39.87), “growth” (32.00), “TGF-beta” (27.11), and “fibrogenesis” (23.05); Cluster 4 emphasizes non-invasive biomarkers and prediction models for managing biliary atresia and liver fibrosis, with terms such as “chronic hepatitis C” (36.61), “stiffness measurement” (34.80), and “platelet ratio index” (29.53); and Cluster 5 highlights noninvasive imaging techniques for diagnosing biliary atresia and fibrosis in pediatric liver disease, including frequent terms like “validation” (38.00), “neonatal hepatitis” (32.50), “sonography” (31.67), and “hepatobiliary scintigraphy” (25.80). The overlay visualization in Figure 7B maps keyword co-occurrence over time, with color indicating the average appearance period of each keyword: yellow nodes represent more recent keywords and emerging research trends, while blue nodes indicate earlier, established topics. Figure 7C, a circular heatmap of the top 50 keywords by frequency (excluding terms like “biliary atresia” and “fibrosis”), illustrates temporal shifts in research focus, with deeper shades indicating higher frequency in specific years and visualizing changes in research emphasis over time. In Figure 7D, CiteSpace highlights the top 14 keywords with the strongest citation bursts from 2000 to 2024, representing significant emerging trends. Notably, several keywords exhibit citation bursts continuing into 2024, such as “pathogenesis” (2015–2024), “outcome” (2015–2024), “elasticity imaging techniques” (2021–2024), and “shear wave elastography” (2021–2022), marking topics that have garnered substantial attention and highlighting current and evolving areas of interest in hepatic fibrosis research within the context of biliary atresia.

### 3.7 Analysis of co-cited references

**Figure 8:**
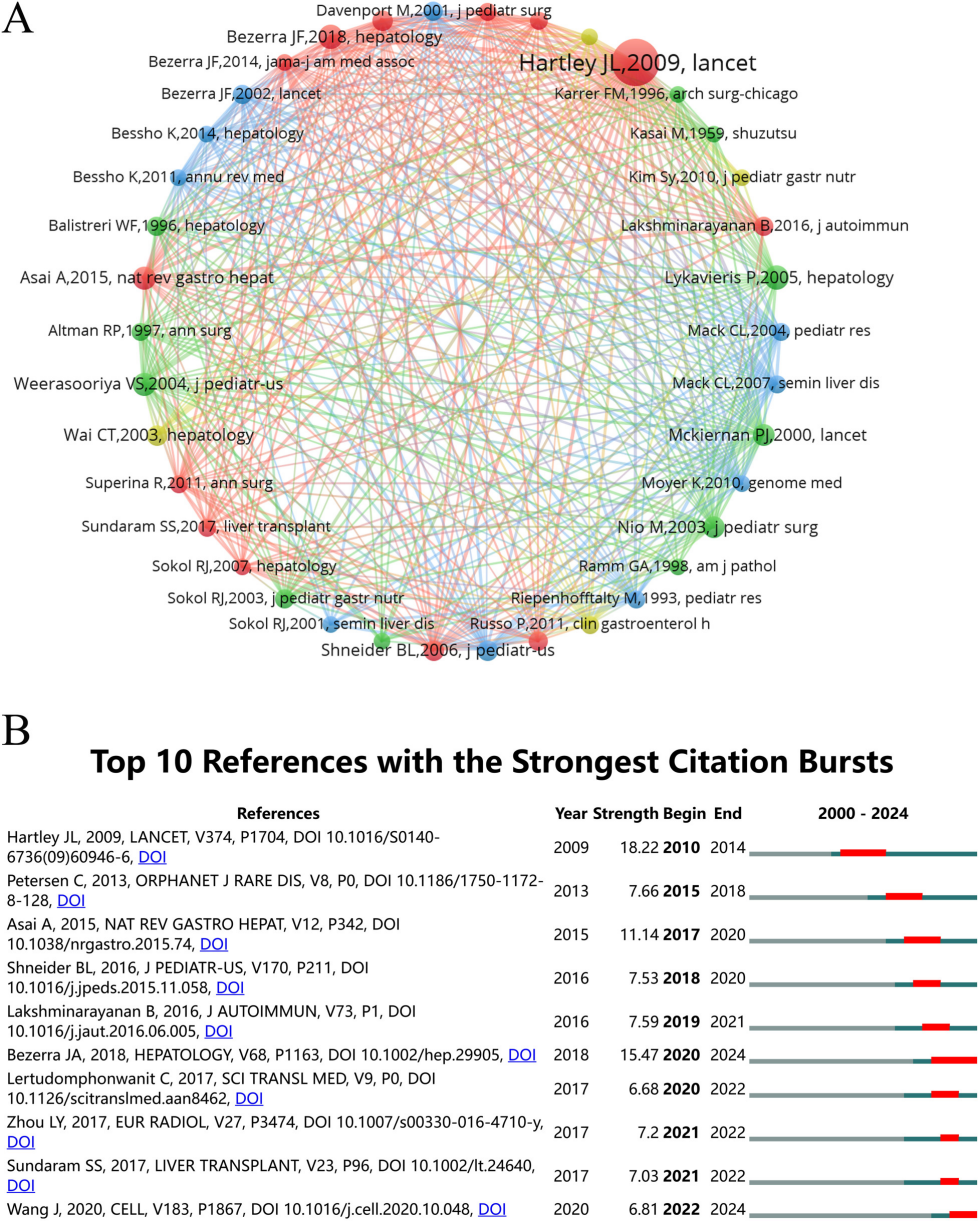
Mapping of co-cited references in studies on liver fibrosis in biliary atresia. **(A)** Co-citation network of references clustered using VOSviewer and Pajek, highlighting influential studies in hepatic fibrosis related to biliary atresia. **(B)** Top 10 references with the strongest citation bursts in hepatic fibrosis research related to biliary atresia from 2000 to 2024.

Co-cited references, collectively cited by researchers, provide essential insights into core research areas within a field. Using VOSviewer, we visualized these references to identify key research themes in hepatic fibrosis related to biliary atresia. Across our dataset, a total of 10,043 references were cited in this research area. By applying a citation threshold of 30 or more, the analysis was refined to include 38 pivotal references, which are organized into three distinct clusters, each represented by a unique color in Figure 8A, with the top five highly co-cited references from each cluster listed in Table 5 to provide a focused overview of key works within these groups. The red cluster focuses primarily on early diagnosis, fibrosis assessment, and surgical outcomes for biliary atresia, with an emphasis on liver function preservation and patient survival. Key studies within this cluster, such as those by Lykavieris (18), Weerasooriya (19), and McKiernan (20), underscore the importance of early intervention in improving long-term prognosis. The green cluster highlights the critical roles of surgical expertise, intervention timing, and genetic factors in shaping patient outcomes. Foundational works in this cluster, including Hartley (21) and Bezerra (22), explore clinical management strategies, while Asai (23) offers a detailed analysis of disease pathogenesis to elucidate underlying mechanisms. The blue cluster investigates the complex pathogenesis of biliary atresia, examining factors such as viral infections, genetic predispositions, and immune responses. Studies by Shivakumar (24) and Bezerra (25) in this cluster analyze immune mechanisms and gene expression that contribute to bile duct obstruction and fibrosis. Together, these clusters capture the central research themes and advances within the field, providing a comprehensive framework for understanding the multifaceted nature of biliary atresia and its clinical implications.

**Table 5:** Top 5 highly Co-cited references for each cluster.

First Author	Year	Journal	Cluster	Citations	Main focus of the literature
Lykavieris et al. (18)	2005	HEPATOLOGY	1	56	Study assesses long-term outcomes of Kasai operation in adult biliary atresia survivors.
Weerasooriya et al. (19)	2004	J Pediatr	1	52	Investigates the correlation between hepatic fibrosis and survival rates in patients with BA.
McKiernan et al. (20)	2000	The Lancet	1	48	Evaluates the incidence and outcomes of biliary atresia surgery in the UK and Ireland.
Nio et al. (63)	2003	Journal of Pediatric Surgery	1	47	Analyzes survival rates and outcomes following surgery for biliary atresia in Japan.
Wai et al. (64)	2003	HEPATOLOGY	1	45	Develops a noninvasive index to predict liver fibrosis and cirrhosis in chronic hepatitis C patients.
Hartley et al. (21)	2009	The Lancet	2	170	Reviews the management, outcomes, and understanding of biliary atresia in infants.
Bezerra et al. (22)	2018	Hepatology	2	59	Addresses clinical challenges and future research directions for biliary atresia in the 21st century.
Asai et al. (23)	2015	Nature Reviews Gastroenterology & Hepatology	2	54	Explores the pathogenesis of biliary atresia to better understand its clinical presentations.
Shneider et al. (65)	2006	Journal of Pediatrics	2	45	Investigates prognostic factors affecting outcomes in biliary atresia children.
Chardot et al. (66)	2013	Journal of Hepatology	2	43	Analyzes outcomes of biliary atresia in France post-liver transplantation availability.
Shivakumar et al. (24)	2004	Journal of Clinical Invest	3	43	Examines IFN-*γ*'s role in bile duct obstruction using a mouse model.
Bezerra et al. (25)	2002	The Lancet	3	39	Explores genetic expression linked to inflammation in biliary atresia livers.
Davenport et al. (31)	2001	Journal of Pediatric Surg	3	38	Investigates immunological patterns in EHBA to correlate inflammation with outcomes.
Mack et al. (57)	2004	Pediatric Research	3	37	Studies CD4+ Th1 cells’ role in portal tract inflammation in biliary atresia.
Narkewicz et al. (67)	1993	Pediatric Research	3	36	Explores fetal liver enzyme induction under chronic hypoglycemia conditions.

In addition to co-citation analysis, citation bursts provide a measure of references that garnered heightened interest over specific time periods, reflecting shifting research priorities. In our study, the top 10 references with the strongest citation bursts were identified using CiteSpace, as illustrated in Figure 8B. Among these, the work by Hartley et al. (21) exhibited the highest citation burst strength of 18.22, sustained from 2010 to 2014, indicating its profound impact and continued relevance within the research community during this period. These findings from co-citation and citation burst analyses highlight the evolving focal points and influential contributions in hepatic fibrosis research related to biliary atresia.

## 4 Discussion

Biliary atresia is the leading cause of pediatric hepatic disease, rapidly progressing to liver fibrosis and failure without timely intervention (26, 29, 30). Understanding the underlying mechanisms, pathological alterations, and clinical impacts of fibrosis is essential for improving diagnostic and therapeutic strategies (27, 28). This study employs bibliometric analysis to evaluate global research trends from 2000 to 2024, highlighting the interplay between liver fibrosis, molecular pathways, and immune responses. It examines publication trends, impact, and contributions from various countries and institutions, providing a comprehensive overview of progress, challenges, and future directions in this field.

The analysis reveals a marked escalation in research activity focused on biliary atresia-associated hepatic fibrosis. From 2000 to 2014, publication rates remained stable (223 publications), reflecting an exploratory phase. In contrast, a paradigm shift occurred post-2015, with publications surging to 296 by 2022, notably peaking between 2017 and 2020. This sharp rise (*χ*^2^ = 113.28, *p* < 0.001) underscores the growing scientific interest and clinical relevance of the field. This surge is linked to three pivotal advancements: first, breakthroughs in non-invasive diagnostics—evidenced by keyword co-occurrence of “serum biomarkers” (e.g., MMP-7, M2BPGi) and “imaging modalities” (e.g., shear wave elastography)—have reduced reliance on liver biopsy; second, progress in molecular pathogenesis, such as biliary epithelial cell dysfunction and aberrant TGF-β/Smad signaling (reflected in “epigenetics” and “immune dysregulation” clusters), has shifted the field toward targeted mechanisms; and third, expanded international collaborations (e.g., U.S.-China partnerships, International Pediatric Hepatology Group trials) have facilitated data sharing. Furthermore, advances in genomics and immunology have deepened mechanistic insights into fibrotic pathology and therapeutic strategies. The high *R*^2^ value (0.9982) confirms the sustained momentum of these initiatives, suggesting that continued investment and interdisciplinary collaboration will yield critical insights for advancing early diagnosis and therapeutic regimens.

This study analyzes high-impact literature to emphasize key features of influential publications. By combining “Highest First Citations,” “Most Relevance,” and “Usage” metrics, a holistic view of academic standing emerges. Intersection analysis revealed 12 pivotal studies. Notable investigations include Davenport et al. (31), who correlated reduced CD68 macrophage and ICAM-1 expression with improved postoperative outcomes; Strazzabosco et al. (32), who identified Notch signaling disruptions that worsen fibrosis; and Rebecca et al. (33), who highlighted the role of epithelial-mesenchymal transition (EMT). Hartley et al. (34) emphasized early diagnosis despite limited fibrosis understanding, while Haafiz (35) stressed the roles of cholestasis and oxidative stress. Bezerra et al. (36) linked gene expression profiles to transplant-free survival. Zheng et al. (26) highlighted the synergistic effects of inflammation and EMT, advocating for targeted therapies. Duan et al. (37) demonstrated that sound touch elastography (STE) is a reliable non-invasive alternative for fibrosis assessment. Kotb et al. (38) identified Kotb disease—a variant caused by congenital aflatoxicosis and GSTM1 deficiency—advocating for early screening. Additionally, Jannone et al. (39) found progressive cellular senescence in BA livers, while Yoeli et al. (40) linked elevated Galectin-3 to advanced fibrosis. Chusilp et al. (5) revealed that viral infection and biliary defects activate hepatic stellate cells, promoting fibrosis. The “Usage (last 180 days)” metric identified 14 publications intersecting with “Most Relevance,” indicating a shift from basic pathophysiological studies to clinical diagnostics, with increasing emphasis on non-invasive techniques.

Liver fibrosis in biliary atresia presents challenges that make international collaboration essential. Key contributors, particularly China, the United States, and Japan, have shown substantial growth. As shown in Figure 3, the U.S. and China lead in publication volume and play a central role in the global network. Centrality analysis (Figure 3B) reveals the U.S. holds a core position, while China and European nations (e.g., UK, Germany) actively contribute to global research. Figure 3C illustrates steady publication increases from these nations, reflecting long-term commitment. The collaboration network analysis in Figure 3D emphasizes strong partnerships, particularly between the U.S. and China, which have been instrumental in driving advancements.

Over the past decade, progress has been significant in East Asia. As China focuses on this area, institutions like Sun Yat-sen University, Capital Medical University, and Shanghai Jiao Tong University have become increasingly involved. Research is driven by three major networks: a U.S.-based consortium (University of Cincinnati and Children's Hospital of Philadelphia); a partnership between Fudan University and the Chinese Ministry of Health; and a Southeast Asian collaboration (Chulalongkorn and Mahidol University). These collaborations facilitate data sharing and innovative strategies. regarding output, Chulalongkorn University and Fudan University have published 39 and 34 papers, respectively, while King's College Hospital (1,221 citations) and the University of Cincinnati are the most influential. This activity correlates with the rising BA incidence in East Asia, leading to increased funding and deeper collaborations.

The productivity distribution aligns with Lotka's law, where a small group of prolific authors drives the field. Notable contributors include Professors Yong Poovorawan, Paisarn Vejchapipat, and Voranush Chongsrisawat (Thailand), and Professor Shan Zheng (China). Poovorawan et al. investigate molecules like Autotaxin and Clusterin to refine diagnostic and prognostic protocols (37–42). Zheng et al. focus on molecular mechanisms involving inflammation and immune responses (43–47). Davenport, Bezerra, and Mack are the most highly cited authors: Davenport et al. have improved surgical outcomes (48); Bezerra et al. investigate gene expression and IL-33 signaling (36, 49); and Mack et al. focus on immune responses and cytomegalovirus infection (50, 51). Network analysis (Figure 5C) identifies two major collaborative entities led by Poovorawan and Zheng, respectively. While geographic proximity facilitates resource sharing, future initiatives should focus on expanding collaborations to ensure diversity and sustainability.

Core journals play a crucial role in advancing this field. According to Bradford's Law, Zone 1 consists of seven major journals. *Pediatric Surgery International*, *Journal of Pediatric Surgery*, and *Journal of Pediatric Gastroenterology and Nutrition* emerge as leaders due to volume and academic impact. *Hepatology* stands out with the highest average citations per article (56.63) and impact factor (12.9). Figure 6C highlights their prominent positions in the co-citation network. Collaborating with these core journals is vital for disseminating high-impact findings.

Keyword co-occurrence analysis reveals research hotspots across five main clusters: etiology, management, molecular mechanisms, non-invasive biomarkers, and imaging. The first cluster focuses on pathogenesis, including viral infections and bile duct toxins. The second emphasizes “prognosis” and “predictive factors” for personalized management. The third examines molecular pathways like “TGF-beta” and biomarkers such as MMP-7 (47). The fourth addresses non-invasive biomarkers like elastography (48), while the fifth investigates imaging techniques, particularly “shear wave elastography,” for improving early detection (52, 53). Analysis of 50 key terms reveals a shift from basic research to clinical applications like liver stiffness measurement. CiteSpace analysis identifies 14 keywords with significant citation bursts, such as “pathogenesis” (2011–2024) and “elastography techniques” (2021–2024), signaling future clinical directions.

Co-citation analysis reveals three thematic clusters. The first (Table 5) emphasizes early diagnosis and clinical outcomes (18–20). The second focuses on surgical expertise and basic pathogenesis, with contributions from Hartley et al. (21) and Bezerra et al. (22) exploring etiology. The third investigates multifaceted pathogenesis involving viral and genetic factors (24, 25, 54, 55). Citation burst analysis highlights the profound influence of Hartley et al. (21) and the emerging trends in molecular advances by Bezerra et al. (22).

In conclusion, both co-citation and citation burst analyses demonstrate the dynamic nature of biliary atresia research, highlighting advancements in pathogenesis, diagnostics, and therapeutics. This provides a solid framework for future research and emphasizes the critical role of interdisciplinary strategies in addressing liver fibrosis in biliary atresia.

## 5 Limitation

Limitations of this study must be acknowledged. First, the analysis relies exclusively on the Web of Science Core Collection, potentially omitting relevant studies from other databases (e.g., Scopus, PubMed). Additionally, our search strategy focused solely on the terms “biliary atresia” and “liver fibrosis,” which may have excluded publications exploring specific therapies such as UDCA or corticosteroid combinations in BA, potentially limiting the analysis of their controversial clinical role. Second, the exclusion of non-article content types (e.g., conference proceedings) and the restriction to publications from 2000 to 2024 may narrow the analytical scope. Third, variability in bibliometric tools employing distinct algorithms could influence outcome interpretation. Geographic bias may further underrepresent regions with limited access to academic journals.

Importantly, while this study elucidates global research trends, it also reveals critical translational gaps: despite decades of investigation and proliferating publications, biliary atresia (BA) remains the leading cause of pediatric liver transplantation, with minimal improvement in clinical outcomes. The disconnect between mechanistic discoveries (e.g., host-environment interaction studies with preventive potential) and therapies that meaningfully alter survival persists, exacerbated by the dominance of repetitive observational studies over hypothesis-driven research. Finally, the focus on quantitative analysis limits opportunities for deeper qualitative insights. Future research should prioritize translational collaboration, expand data sources to include diverse publication types, and integrate mixed-method approaches to address these unmet needs.

## 6 Conclusion

In conclusion, significant advancements have been achieved in the exploration of liver fibrosis associated with biliary atresia (BA) over recent decades, particularly concerning early diagnosis, molecular mechanisms, non-invasive diagnostic technologies, and international collaboration. Research trends reveal an increasing interest in BA-related fibrosis, particularly in nations such as China, the United States, and Japan. Key journals have played an integral role in disseminating knowledge and propelling research forward. Analyses of high-impact literature have illuminated pivotal research trajectories and major breakthroughs within the field. Despite the inherent limitations in data sources and methodologies, this study offers valuable insights for future research on BA-related liver fibrosis, underscoring the necessity of interdisciplinary cooperation and a focus on emerging research domains.

## Data Availability

The original contributions presented in the study are included in the article/Supplementary Material, further inquiries can be directed to the corresponding author.
